# Porous Si-SiO_2_ UV Microcavities to Modulate the Responsivity of a Broadband Photodetector

**DOI:** 10.3390/nano10020222

**Published:** 2020-01-28

**Authors:** María R. Jimenéz-Vivanco, Godofredo García, Jesús Carrillo, Francisco Morales-Morales, Antonio Coyopol, Miguel Gracia, Rafael Doti, Jocelyn Faubert, J. Eduardo Lugo

**Affiliations:** 1 Semiconductor Devices Research Center, ICUAP, BUAP, Ciudad Universitaria, Puebla Pue. C.P. 72570, Mexico; jimenezvmr10@gmail.com (M.R.J.-V.); godgarcia@yahoo.com (G.G.); jecarril@siu.buap.mx (J.C.); acoyopol@gmail.com (A.C.); 2 Optics Research Center, A.C., Loma del Bosque 115, Col. Lomas del Campestre León, León C.P. 37150, Gto, Mexico; fcomm9@gmail.com; 3 IFUAP, Benemérita Universidad Autónoma de Puebla, Ed. IF2, Col. San Manuel, Puebla C.P. 72570, Mexico; gracia@ifuap.buap.mx; 4 Faubert Lab, School of Optometry, University de Montreal, Montreal, QC H3T 1P1, Canada; rafael.doti@gmail.com (R.D.); faubert.umontreal@gmail.com (J.F.)

**Keywords:** photonic nanoscience, nanotechnology, porous Si-SiO_2_, UV filters, responsivity

## Abstract

Porous Si-SiO_2_ UV microcavities are used to modulate a broad responsivity photodetector (GVGR-T10GD) with a detection range from 300 to 510 nm. The UV microcavity filters modified the responsivity at short wavelengths, while in the visible range the filters only attenuated the responsivity. All microcavities had a localized mode close to 360 nm in the UV-A range, and this meant that porous Si-SiO_2_ filters cut off the photodetection range of the photodetector from 300 to 350 nm, where microcavities showed low transmission. In the short-wavelength range, the photons were absorbed and did not contribute to the photocurrent. Therefore, the density of recombination centers was very high, and the photodetector sensitivity with a filter was lower than the photodetector without a filter. The maximum transmission measured at the localized mode (between 356 and 364 nm) was dominant in the UV-A range and enabled the flow of high energy photons. Moreover, the filters favored light transmission with a wavelength from 390 nm to 510 nm, where photons contributed to the photocurrent. Our filters made the photodetector more selective inside the specific UV range of wavelengths. This was a novel result to the best of our knowledge.

## 1. Introduction

Porous silicon (PS) is a promising material for many different applications, such as solar cells, specifically as anti-reflection coating [1], chemical sensing [2,3], biomedical applications [4], biosensing [5,6], as a photodetector [7], or light-emitting diode [8]. The anti-reflection coating (ARC) reduces reflection losses thereby increasing the photocurrent and efficiency in solar cells [9,10,11,12]. A microcavity filter (MF) obtained in the IR range has been used as the ARC, where the maximum transmission at the localized mode wavelength was 95.2%, with a minimum reflectance of 4.8% at the same wavelength. The PS ARC was deposited onto a solar cell, where the entire surface of the solar cell was covered, and its external quantum efficiency (EQE) was measured, with and without the MF. The EQE at different wavelengths matched the shape of the MF transmission spectra [9]. ARCs are used to convert down the higher energy solar radiation into a wide range of light spectra, which is absorbed more efficiently into bulk Si [10]. In another interesting application, silver nanoparticles were infiltrated within a porous silicon photonic crystal to detect the trace of explosives (Rhodamine 6G dye and Picric acid explosives) using surface-enhanced Raman scattering [13]. Recently, porous silicon nanoparticles (PSN) have been used for the tunable delivery of camptothecin, a small therapeutic molecule, where PSN acted as a therapeutics nanocarrier into the electrospun composite of poly fibers [14]. Additionally, mesoporous Si has been investigated for applications in biology and medicine, and an example is a porous silicon container employed to enclose the drug and release it in a controlled manner [15]. Previously, PS MFs have been fabricated to work as red-infrared filters coupled with a silicon photodetector, where the localized mode can be tuned by changing the optical path of the defect layer [16]. The reflectivity at the localized mode wavelength also increases. This effect occurs because PS has less absorption at long wavelengths [16,17]. This kind of filter can be lifted off from the Si substrate creating a free-standing PS MF. This MF can be transferred to another substrate such as quartz and glass [18,19]. Infrared long-wave pass and short-wave pass filters based in macroporous Si have been investigated [20]; they can be used together to suppress the radiation from shorter wavelengths. Consequently, the signal-to-noise performance of detectors may improve [18]. Moreover, luminescent silicon quantum dots embedded in free-standing PS and PS MFs infiltered with CdSe/ZnS and AgInS_2_/ZnS quantum dots have been reported [21,22,23]. The localized mode of the PS MF was tuned experimentally to match the emission spectra of CdSe/Zn and AgInS_2_/ZnS quantum dots to achieve enhanced photoluminescence. The authors mentioned that the localized mode of the MF modulated the photoluminescence of the quantum dots [22,23]; where electrical and thermal tuning of localized modes could also be achieved by infiltrating liquid crystals in PS MFs [24]. PS was also utilized as an anode for fast charge-discharge in lithium-ion batteries [25,26], and it was found that laser carbonization and wet oxidation PS structures had memristive properties. The first memristor showed properties of plasticity and short/long term memory, whereas the second exhibited strong filamentary-type resistance switching; and they have been used as two terminal resistive memory cells [27].

As the reader already noticed, PS is a versatile material, and herein, we will focus on the optical properties of PS, which allow the design of various kinds of interference filters in a broad range of wavelengths. Among these designs, we found rugate filters [28,29], Fibonacci filters [30], Bragg reflectors filters (BRF) [31,32], and MFs [33]. However, many drawbacks have been found in these filters, such as high chemical instability, high photon losses due to light absorption, and scattering in the visible and UV ranges. Some solutions to these problems have been identified by carrying out dry oxidation in PS structures [32,34], and recently it has been possible to manufacture porous Si-SiO_2_ filters (BRF and MF) in the UV range [34,35,36]. Our specific goal is integrating a PS MF in a photodetector to enlarge the responsivity spectrum bandwidth. In the past, the integration of different PS bandpass filters in Si photodetectors was attainable to achieve different filtering effects. It was possible to tune the responsivity spectrum from green to the near-infrared range obtaining all-Si color-sensitive photodetectors in that wavelength interval but with no below the green range [16,31,37]. It has been reported that UV filters based in multilayer stacks show substantial drawbacks in the deep UV spectral range due to the limited number of transparent materials within that range. They have been proposed as an alternative to extending optoelectronic technology towards the UV spectral range; for instance, PS filters based on macroporous silicon and low-pressure chemical vapor deposition were tested for many solar-blind applications such as electrical spark imaging and non-line of sight (NLOS) UV optical communications [18,38]. Following the same line of thought of extending silicon-based selective filters towards the UV spectral range; in this work, porous Si-SiO_2_ microcavities were used to modulate the responsivity of a broad photodetector with a detection range from 300 to 510 nm. The porous Si-SiO_2_ microcavities made the photodetector more selective in the UV range; and all microcavities had a localized mode close to 360 nm in the UV-A range. The maximum transmission at the localized mode wavelength (360 nm) was dominant in the UV-A range, allowing high energy photons to pass and produce a maximum peak in the photodetector.

This work is divided as follows: In Section 2, we give some technical details for materials and methods. Section 3 describes the results and their discussion. Finally, in Section 4, we present some conclusions.

## 2. Materials and Methods

### 2.1. Porous Silicon Microcavities Filters

PS microcavities filters were fabricated using p-type Si wafers, with (100) orientation and electrical resistivity between the range 0.01–0.02 Ω cm. The microcavities were etched anodically in an aqueous electrolyte based in HF and ethanol with a volume ratio of 1:1. Before etching, the Si wafers were cleaned with HF and ethanol for 5 min to remove the native oxide. A Keithley 2460 current source controlled by a laptop was employed to deliver a current profile for the microcavities formation, where the current profile consisted of switching two different current pulses, with each current pulse producing low/high porosity layers that respectively corresponded to the low/high refractive index layers. This process produced a stack of layers with specific refractive indexes, while anodization time determined the thickness of the layers. A pause of 3 s was introduced between each current pulse to prevent any porosity gradient.

Anodization etching included low/high current pulses of 5 mA/cm^2^ and 80 mA/cm^2^ to obtain layers with porosities of 39% and 64%, respectively. Meanwhile, the anodization times of 4.1 and 1.1 s were applied to form the first and second porous layers, and finally a third porous layer (defect) was built-in using a current pulse of 80 mA/cm^2^ for 2.2 s. The PS microcavities filters were detached from the Si substrates using the same aqueous electrolyte before mentioned, but a high current density (450 mA/cm^2^) was applied for 2 s. This process created a free-standing PS microcavity filter, which was transferred to a quartz substrate. All samples were rinsed with ethanol and dried at room temperature after the anodization process.

### 2.2. Dry Oxidation in PS Microcavities Filters

Microcavities filters were systematically oxidized using two stages of dry oxidation. In the first stage, a temperature of 350 °C for 30 min was used. In the second stage the oxidation temperature was increased to 900 °C for 1 h. During the oxidation process, the oxygen flow was changed from 1.15 to 4.52 SLPM (standard liter per minute) to observe any change in the microcavity optical response. The reasons behind the two dry oxidation steps were: the first step was a low-temperature pre-oxidation needed to equilibrate the silicon structure. That is, this step prevented the aggregation of the pores during further treatments at higher temperatures. The second step was applied to grow an oxide layer of greater thickness than that obtained in the last oxidation step. The layer thickness is higher than the natural native oxide grown in the environment resulting in the consolidation of SiO_2_.

Transmittance and reflectance spectra measurements were carried out before and after dry oxidation with a Varian (Agilent Technologies, CA, USA) UV-Vis-NIR spectrophotometer at normal incidence, and 20° from 200 to 800 nm. The cross-section SEM images of an oxidized microcavity was obtained using a JEOL-JSM7600F (Jeol, MA, USA).

### 2.3. Photocurrent Modulation by Porous Si-SiO_2_ Microcavities Filters 

**Figure 1:**
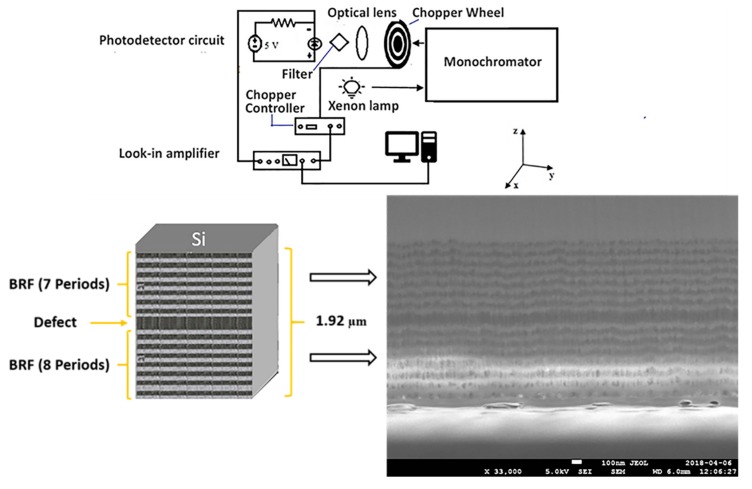
Experimental set-up used to obtain the responsivity of a commercial photodetector with different porous silicon (PS) microcavity filters (up panel). The bottom panel shows the scheme and cross-section SEM images of an oxidized microcavity filter with a total thickness of 1.92 μm.

The photodetector employed to modulate its responsivity was a GVGR-T10GD (Electro Optical Components Inc., CA, USA) based in indium gallium nitride with a spectral detection range from 300 to 510 nm. In the experimental process, an optical chopper SR540 (Stanford Research Systems Inc., CA, USA) with a frequency of 287 Hz was used to cut off the light coming from the Xenon lamp 6254 (Oriel Corporation, CT, USA), concentrated at the input of a monochromator. A lens was used to focus the output light from the chopper, on the photodetector. The PS microcavity filter was placed before the photodetector, which was polarized with 5 volts using a Keithley 2460 source (Keithley, OHIO, USA), and then the photodetector photocurrent inputted a Look-in amplifier SR-530 (Stanford Research Systems, CA, USA)). The filtered and amplified photocurrent versus light wavelength (from 276 to 536 nm) was displayed in a PC. The experimental set-up is shown in Figure 1.

### 2.4. Theoretical Mechanism to Modulate the Responsivity of a Broadband Photodetector in the UV Optical Range with Porous Si-SiO_2_ Microcavities

In an ideal photodetector, its responsivity is proportional to the input light power being the proportional factor, the so-called quantum efficiency η, which is a function of the light reflection *R* at the surface, the fraction of electron-hole pairs that contribute effectively to the photocurrent ζ, the light absorption in the bulk of the material α, the photodetector depth *d*, and the light wavelength.

Herein, we proposed to modulate the responsivity of a commercial photodetector by changing *R*. This was done by adding a microcavity filter, where the spectral reflection response of the microcavity filter modifies the original reflection spectral response at the surface of the commercial photodetector completely. The microcavity filter reflection depends on the high and low refractive indexes and thickness values, as well as the refractive index and thickness of the defect layer values. Initially, the microcavities are made of PS to filter out blue light. This means that the layers’ refractive index values, controlled by the porosities and in which the crystalline silicon nanostructures remains, were chosen to respond in that particular region of the light spectrum. However, our primary goal was to achieve responsivity modulation within the UV region using a silicon-based filter. We cannot use the sensitive blue light microcavities for that purpose because the crystalline silicon nanostructures strongly absorb light within the UV and blue regions. This absorption process is equivalent to having a 100% reflection at the photodetector surface. Thus, the quantum efficiency, and consequently, the responsivity, tends to zero. The oxidation process helps to avoid this problem in two ways: first, since there is a phase change, from crystalline silicon to silicon dioxide, the layers’ refractive index values decrease, pushing the filter response towards the UV region. Second, there is much less light absorption in the UV band. The oxygen flow value determines the concentration of silicon dioxide, followed by the layer’s refractive index values, and consequently, the spectral response of the filter. More specifically, the filters spectral response contains a unique photonic state known as a localized state represented as a resonant peak in the reflection spectrum. It is a state that drastically modifies the responsivity, and therefore, it is the flow of oxygen that determines the final position of the localized state along with its reflection amplitude.

## 3. Results and Discussion

### 3.1. Porous Silicon Microcavities Filters in the Blue Range

**Figure 2:**
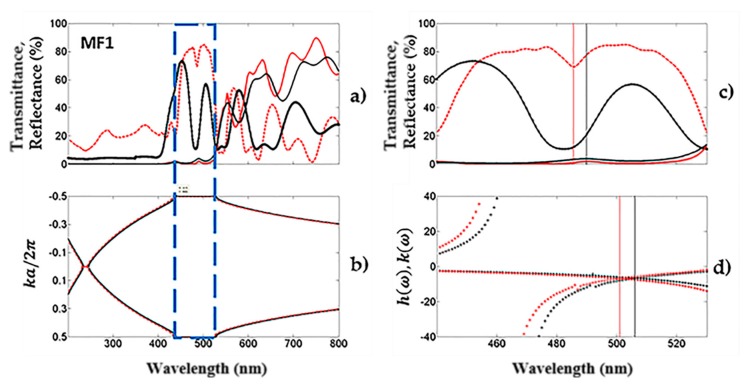
Experimental averaged transmission and reflection spectra of four microcavities compared to its theoretical spectra. (**a**) The theoretical (red line) and experimental (black line) averaged reflection and transmission spectra for four unoxidized microcavities obtained in the blue optical range; (**b**) the averaged band structure of the same microcavities. (**c**,**d**) are the comparison of the averaged theoretical and experimental defect mode locations. In (**d**), two angles of incidence (zero and 20 degrees) were considered.

The averaged theoretical (red line) and experimental (black line) reflection (broken line) and transmission (solid) spectra from the PS microcavities filters are depicted in Figure 2a. This corresponded to the average measurements of transmittance and reflectance spectrum from four microcavities. All four MCs were designed to show a localized mode in the same wavelength (590 nm). Experimental transmission and reflection measurements of the microcavities were taken using a UV-VIS-NIR spectrophotometer in the wavelength range from 200 to 800 nm. The transmission spectrum was measured at normal incidence and reflectance spectrum of the same microcavities was obtained at 20°. Meanwhile, theoretical transmission and reflection spectrum were obtained using the matrix method. This method is well known (Supplementary Materials) for obtaining the theoretical spectrums that we considered the angle measurements taken with the UV-VIS-NIR spectrophotometer.

The theoretical reflection spectrum of the microcavity predicts more reflection in the UV range than its experimental counterpart, while the theoretical and experimental transmission spectra matched very well. Moreover, the light transmission was low in the UV-VIS range due to strong absorption of Si. The localized mode of the microcavity was located at 490 nm and exhibited a maximum transmission peak of 4%, while that in the visible range from 555 to 800 nm showed high light transmission. This result was because PS had small absorption losses in this range of the electromagnetic spectrum, where the primary photon loss was light scattering. When the reflection spectrum of the microcavity was taken at 20°, the position of the localized mode changed, and it shifted towards high energies (483 nm). It could also be observed in the theoretical reflection spectrum. Figure 2b shows the averaged photonic bandgap (PBG) of an unoxidized microcavity. It was obtained using the dispersion relation inside the first Broullin zone. We calculated it only in one dimension and both measurement angles were considered, which were mentioned before.

We obtained the defect frequency can be obtained using a combination of the transfer matrix technique and variational methods. The defect modes are represented by the maximum peak of transmission in the microcavities [39,40]. When the symmetry of the photonic structure is broken up by a defect layer, it is possible to have a confined state, where photons are trapped and cannot escape, being confined inside the bandgap. We calculated the defect mode frequency for an antisymmetric state because our photonic structures were built as follows:

$${\left(HL\right)}_{8}L{\left(HL\right)}_{7}$$

Our antisymmetric photonic structures based on PS and porous Si-SiO_2_ consisted of 31 layers, alternating between high refractive and low refractive indexes, with a low refractive index defect layer, and where the structure was surrounded by air.

The averaged theoretical defect mode frequency location is shown in Figure 2d, where it is compared with the averaged transmission and reflection spectra (Figure 2c). The defect mode is shown as a transmission maximum or as a reflection minimum at a specific wavelength between the photonic bandgap (PBG) edges. The variational method defect mode location was 18 nm off, concerning the experimental result from the reflection measurements, which was taken at 20 degrees. In this case, the defect mode location is depicted in Figure 2d (red broken line). Using the same variational method, the defect mode location was 16 nm off for the experimental transmission spectrum, which was taken at normal incidence. The localized mode is shown in Figure 2d (black broken line). The transfer matrix method predicted the same position as the experimental result. Both theoretical methods showed good agreement with the experiments.

### 3.2. Porous Si-SO_2_ Microcavities Filters in the UV

**Figure 3:**
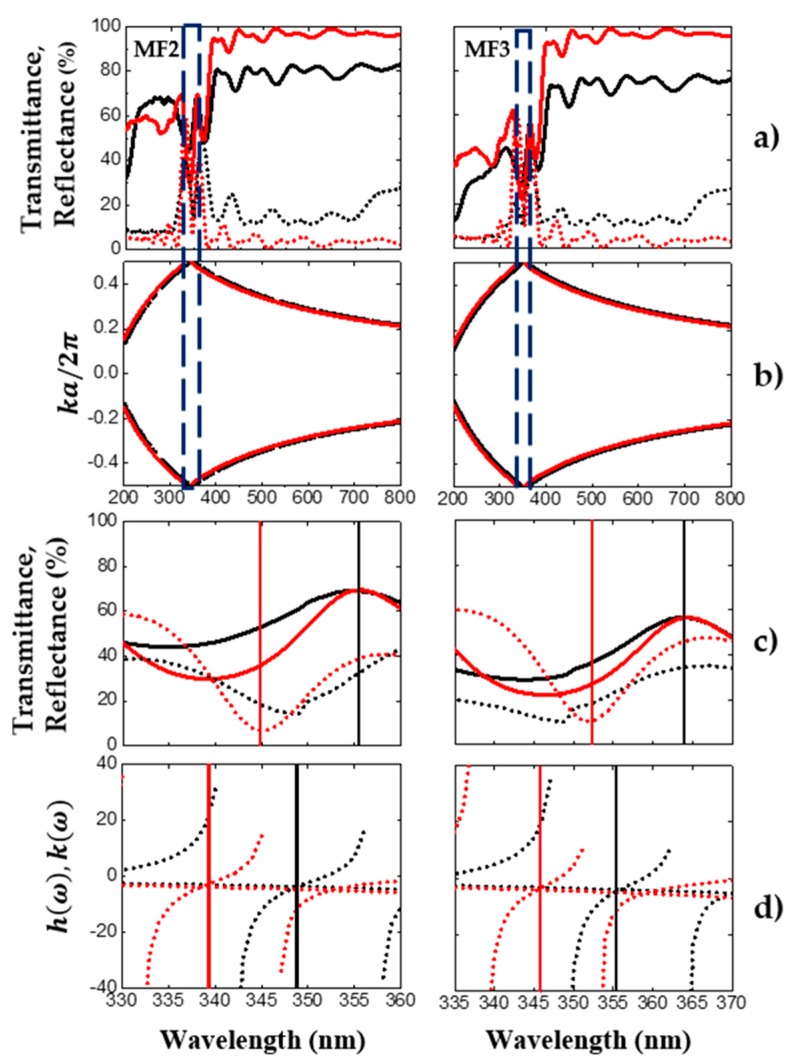
The averaged theoretical and experimental transmission and reflection spectra of two UV microcavities filters, with the averaged photonic bandgap and defect mode locations. (**a**) The averaged theoretical (red line) and experimental (black line) reflection (broken line) and transmission (solid line) spectra of oxidized microcavities obtained in the UV range; (**b**) the averaged photonic bandgap structure (empty box) of the microcavities. (**c**,**d**) show the comparison of the averaged theoretical and experimental defect mode locations. In (**d**), two angles of incidence (zero and 20 degrees) were considered.

Averaged transmission (solid line) and reflection (broken line) spectra from porous Si-SiO_2_ microcavities filters are depicted in Figure 3a. The theoretical (red line) and experimental (black line) spectrum showed a good fit. The theoretical spectra were obtained by the transfer matrix (Supplementary Materials) considering both measurement angles, where one was 0°, which corresponded to the transmission measurements, and the other was 20°, considered for the reflection measurements. The microcavity to the left was first oxidized at 350 °C for 30 min and then at 900 °C for 1 hr, and the oxygen flow was 1.15 SLPM (standard liter per minute). The microcavity shown to the right was oxidized using the same temperature and oxidation time mentioned above. In this case, an oxidation flow of 2.21 SLPM was applied. The microcavity MF2 exhibited a maximum transmission amplitude of 70%, and the microcavity MF3 showed a maximum transmission amplitude of 57% in the localized mode wavelength, which indicated that the microcavity MF3 had more absorption losses than the microcavity MF3. Porous Si-SiO_2_ microcavities filters in Figure 3a show a transmission maximum peak (356 and 364 nm) and a reflection minimum peak (349 nm) in the UV range. We observed a mismatch between both peaks because the measurements were taken at different incidence angles.

Figure 3c shows the averaged transmission and reflection spectrum from UV filters compared to the defect mode location obtained with the variational method (Figure 3d). The defect mode location corresponding to the reflection spectrum was 4 nm off using the variational method, which is shown in Figure 3d (red broken line). The position of the defect mode obtained from the variational method (black broken line, Figure 3d) was 9 nm off for the transmission spectrum, as compared to what was predicted using the transfer matrix method.

**Figure 4:**
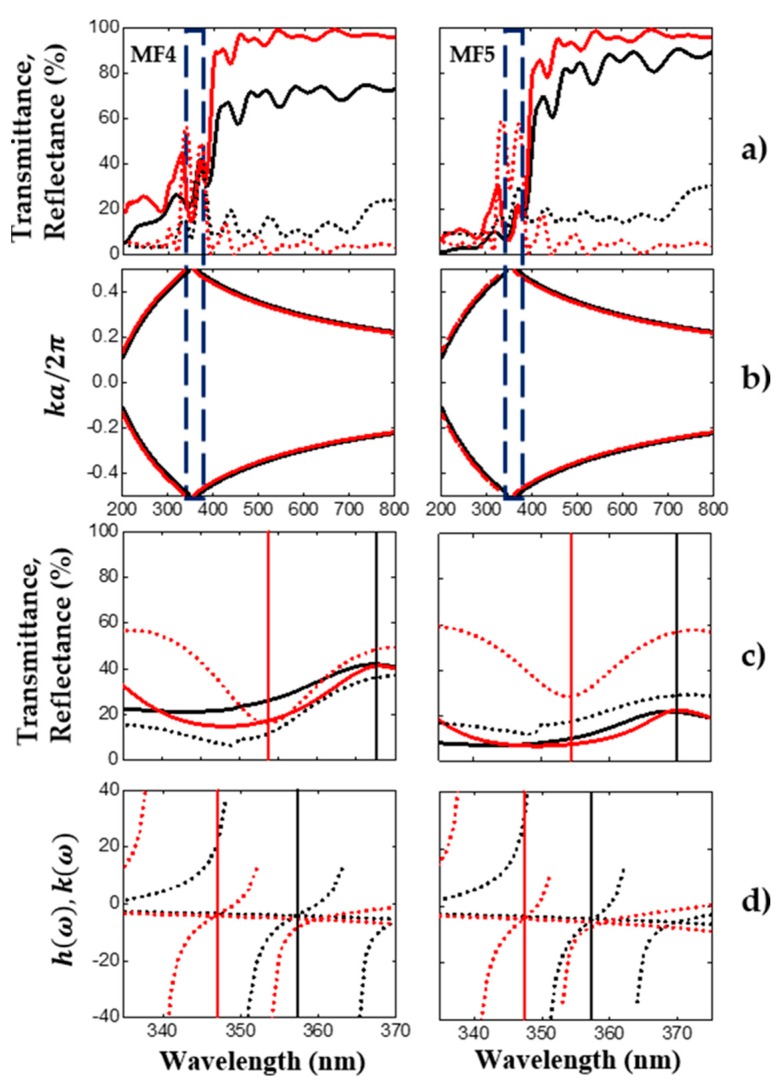
The averaged theoretical and experimental reflection and transmission spectrum of another two UV microcavities filters. Additionally, its averaged photonic bandgap and the defect mode locations are depicted. (**a**) The averaged theoretical (red line) and experimental (black line) reflection (broken line) and transmission (solid line) spectra of oxidized microcavities obtained in the UV range; (**b**) the averaged photonic bandgap structure (empty box) of the microcavities. (**c**,**d**) are the comparison between the averaged theoretical and experimental defect mode locations. In (**d**), two angles of incidence (zero and 20 degrees) were considered.

Figure 4a depicts the averaged theoretical (red line) and experimental (black line) transmission (solid line) and reflection (broken line) spectra of two oxidized MF. In this case, the porous Si-SiO_2_ microcavity filter MF4 showed a maximum transmission peak (368 nm) in the UV range, with an amplitude of 42%. Meanwhile, the microcavity MF5 displayed a maximum transmission peak at 369 nm, with an amplitude of 21% (red and black solid lines). A reflection minimum peak (349 nm) is also shown in both microcavities (red and black broken lines). The maximum transmission peaks were less intense than the microcavities shown in Figure 3. Here, the same temperature and oxidation time were employed to obtain the UV filters, but the oxygen flow applied for sample MF4 was 3.39 SLPM, while for sample MF5, it was 4.52 SLPM. The oxygen flow was increased gradually.

Figure 4c,d shows the averaged theoretical (red line) and experimental (black line) reflection (broken line) and transmission (solid line) spectra near to the defect mode location. The variational method was employed to obtain the defect mode location between the PBG edges. The broken red plots (Figure 4d) corresponded to the defect mode position of the microcavities measured at 20°. The defect mode locations of microcavities measured at normal incidence are depicted as broken black lines in Figure 4d. There was a mismatch between the defect mode position calculated using the transfer matrix and the variational method.

### 3.3. Refractive Index of Porous Silicon and Porous Si-SiO_2_

**Figure 5:**
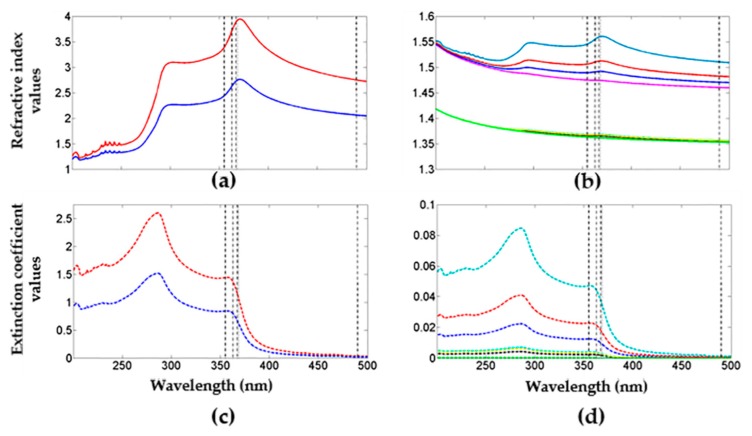
Theoretical values of the complex refractive index for PS layers and porous Si-SiO_2_ layers. (**a**,**b**) shows the refractive index values for PS and porous Si-SiO_2_, and (**c**,**d**) depicts the extinction coefficient values of PS and Porous Si-SiO_2_, respectively, obtained from both models.

The complex refractive index (refractive index and extinction coefficient) of the PS was obtained using the effective medium approximation Maxwell–Garnett. For porous Si-SiO_2_, we used a three-component model developed from the J.E. Lugo model. Figure 5a,b shows the refractive index values, and Figure 5c,d depicts the extinction coefficient values of PS and Porous SiO_2_, respectively, obtained from both models. The continuous lines in Figure 5a correspond to a high refractive index (red line) and a low refractive index (blue line) for PS. Their extinction coefficients are displayed with dotted lines (red and blue) in Figure 5c, where these values were used to fabricate PS microcavities filters in the blue range. The continuous lines of different colors in Figure 5b between 1.45 and 1.6, and the green line with values between 1.35 and 1.5, represent high and low refractive indexes for the porous Si-SiO_2_ layers (see Figure 5b). Their corresponding extinction coefficients are shown below the value of 0.1 (dotted lines) in Figure 5d. The vertical black dotted lines that intersect the complex refractive index components (Figure 5) indicate the exact values utilized to design MF in the blue and UV range.

Figure 5 shows the refractive index and extinction coefficient are high when the PS is not oxidized. Meanwhile, the complex refractive index component values for porous Si-SiO_2_ are lower than the PS values. The growth of SiO_2_ inside PS layers decreased the refractive index and extinction coefficient. Moreover, during the PS layers’ oxidation process, some air and Si fractions were replaced by SiO_2_, causing a lattice expansion and a decrement on the layer’s porosity, as shown in Table 1.

Knowing the refractive index of each layer that made up the microcavity allowed estimation of the theoretical thickness of each layer by fitting the theoretical transmission and the reflection spectrum (applying the matrix method) with its experimental result. The thickness values displayed in Table 1 correspond to theoretical thickness for different microcavities. On the other hand, an increase in the thickness for the oxidized microcavities (MF2, MF3, MF4, and MF5) was shown after dry oxidation compared to the thickness of the unoxidized microcavity (MF2).

Additionally, the thickness of an oxidized microcavity was obtained using SEM measurements. Figure 1 (down panel) shows where the porous Si-SiO_2_ UV filter has a total thickness of 1.92 µm and it can observe a defect between two BRF. The light gray thickness ($d_{H}=48\;\mathrm{nm}$) corresponds to layers with high refractive index (low porosity) and the dark gray thickness ($d_{H}=75\;\mathrm{nm}$) depicts the layers with low refractive index (high porosity). Meanwhile, the defect in the microcavity had twice the thickness of the high porosity layer. The thickness found using both methods showed a difference of a few nanometers, and both results give an approximate thickness value for each layer of an oxidized microcavity filter.

Furthermore, the optical path was modified by decreasing the refractive index and increasing the physical thickness. Finally, the vertical black dotted lines that intersected with the complex refractive index components (Figure 5) indicated the exact values utilized to design MF in the blue and UV range.

### 3.4. Optical Losses Due to Light Absorption

**Figure 6:**
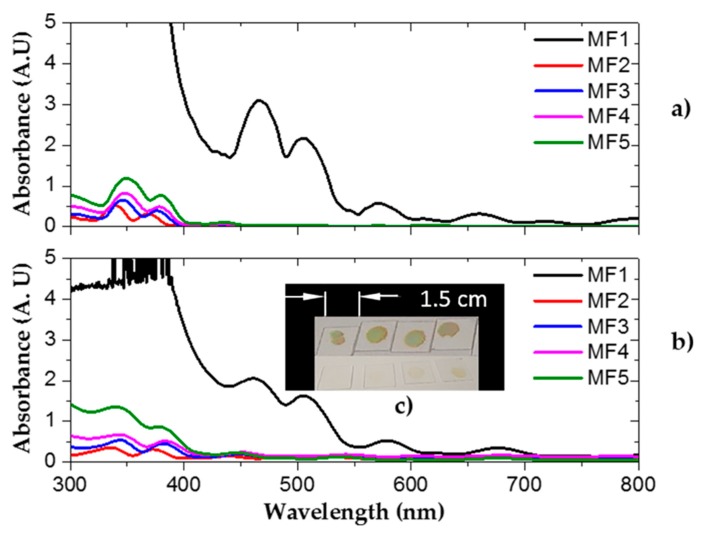
Theoretical (**a**) and experimental (**b**) absorption spectra of MFs in the blue and UV range. (**c-up**) Four unoxidized PS microcavities on a quartz substrate. (**c-bottom**) Four oxidized porous SiO_2_ microcavities.

Optical losses in the VIS and UV range due to light absorption are found frequently in PS. This absorption loss impedes the fabrication of PS filters in the UV range, but these losses can be decreased if the PS structure is thermally oxidized in an oxygen environment [35,36]. Figure 6 (MF1) shows the theoretical (a) and experimental (b) absorbance spectrum of five microcavities. As observed, the absorption was dominant in the VIS and UV range, while in the infrared range, the absorption did not play an important role. It has been reported that optical losses are mainly due to dispersion in the infrared range [41,42]. Moreover, Figure 6a (MF2-MF5) shows the theoretical absorption spectra of four oxidized PS MCs. Figure 6b displays the experimental absorption spectra of the same four oxidized PS MCs filters using different oxygen flows. These plots showed an absorption increase when the oxygen flow builds up. The average absorbance spectra of the oxidized microcavities showed less optical losses due to absorption. Their amplitude decreased more than 70% in the UV range and almost disappeared within the VIS range after dry oxidation. We obtained a good fit between the theoretical and experimental absorption spectra in the VIS and UV range.

These results showed that the defect mode of the PS microcavities filters had a wavelength shift of 134 nm to lower wavelengths when the PS microcavities were oxidized. It also had a wavelength shift of 13 nm to lower wavelengths when a maximum oxygen flow of 4.52 SLPM was applied. Increasing the oxygen flow made the porous Si-SiO_2_ microcavities exhibit less amplitude in the transmission and reflection spectrum inside the UV range. High oxygen flow did not allow oxygen particles to incorporate into the PS structure. Thus, there was less SiO_2_ formation and consequently, optical absorption increased. Besides, a decrease of the PBG bandwidth was achieved by incorporating SiO_2_ within the PS microcavities. This bandwidth decrease happened because there was less contrast between the high refractive index and the low refractive index of the porous Si-SiO_2_ layers.

### 3.5. Photocurrent and Responsivity Measurements of a Commercial Photodetector without and with Filters

**Figure 7:**
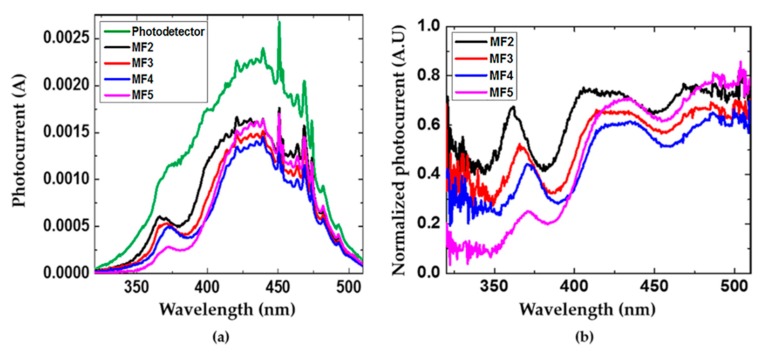
Photocurrents spectra of a commercial photodetector with and without porous Si-SiO_2_ microcavity filters. (**a**) The photocurrent spectra of a commercial photodetector (green line) and its modulated photocurrent with porous Si-SiO_2_ microcavities filters (black, red, blue, and pink lines) and (**b**) the normalized photocurrent.

Porous Si-SiO_2_ UV microcavities filters were used to modulate the photocurrent of a broad photodetector (GVGR-T10GD) with a detection range from 300 to 510 nm. Figure 7 depicts the measured photocurrent of a commercial photodetector (gray line) without and with filters (black, red, blue, pink lines). The MF produced a photocurrent maximum in the UV range, which corresponded to the localized mode of the porous Si-SiO_2_ microcavity filters positioned between 356 and 364 nm. This meant that porous Si-SiO_2_ filters cut off the photocurrent range from 300 to 350 nm. Therefore, in the short-wavelength range, the photons were absorbed and did not contribute to the photocurrent, whereas in the VIS range, photons with wavelength light from 390 to 510 nm were allowed to pass and they contributed to increasing the photocurrent. Besides, all photocurrent spectra modulated with porous Si-SiO_2_ microcavities showed a decline in the UV range, which was attributed to the transmission amplitude of the microcavity as it decreased when the oxygen flow increased.

Figure 7b shows the normalized photocurrents of a commercial photodetector modulated with porous Si-SiO_2_ microcavities filters. We observed that the photocurrent peak at the localized mode wavelength decreased when the filters with less transmission amplitude were used.

**Figure 8:**
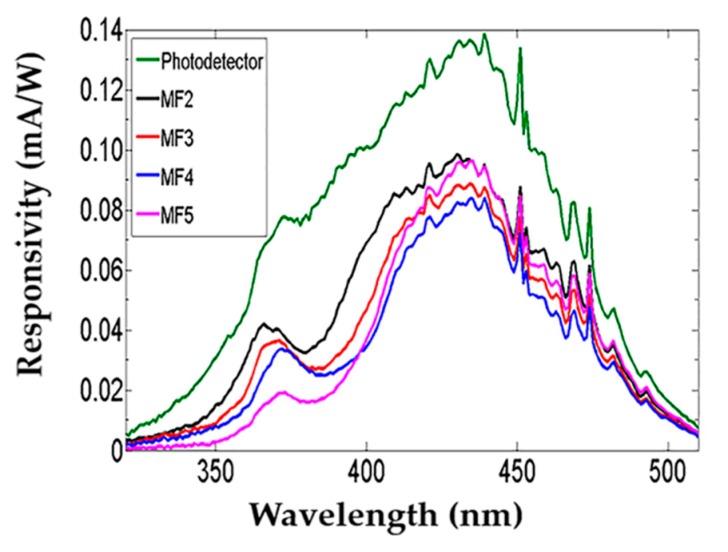
Spectral responsivity comparison of a commercial photodetector without (line green) and with porous Si-SiO_2_ filters (black, red, blue, and pink lines).

Spectra responsivities were obtained using a 150 W Xenon arc lamp as a light source and a monochromator with a light wavelength from 300 to 510 nm at 5 V applied bias. Thus, we calculated the responsivity from:

(1) $$R=\frac{I_{\mathrm{ph}}}{P_{\mathrm{inc}}}$$

where R is the responsivity of the modulate photodetector with porous Si-SiO_2_ microcavities filters, $I_{\mathrm{ph}}$ is the photocurrent in Ampers, and $P_{\mathrm{inc}}$ is the power of the Xenon lamp in watts. The photodetector responsivities without and with porous Si-SiO_2_ microcavities filters were found in the range from 300 to 510 nm. The result is shown in Figure 8.

The spectral responsivity of a commercial photodetector was modified using different porous Si-SiO_2_ filters. It decreased when porous Si-SiO_2_ filters with low transmission in the UV range were placed on the commercial photodetector. However, a maximum peak of detection corresponding to the localized mode of the microcavity was observed in the UV-A range. In the VIS range, an increase of the responsivity was displayed using the MF5 filter. It was due to an increase in its transmission. The spectral responsivity shape was the same as the photocurrent. We also observed that the sensitivity of the photodetector with a filter was lower than the photodetector without a filter.

PS Bragg reflectors and microcavities were used to filter incident light reaching the Si photosensitive wafer. To tailor its spectral response, they were designed to different wavelengths and integrated above the p-n junction of a silicon photodiode. In this way, they were converted in an array of PS detectors sensitive to the color [16,31], where the sensitivity peak could be tuned along from green to near-infrared, and where sharp peaks in the spectral responsivity were achieved using microcavities and color-sensitivity by Bragg-reflectors [16,43]. The transmittance of the PS filters mainly modulates the spectral responsivity of the silicon photodiode. Therefore, when a Bragg reflector is used to modulate the spectral response of the Si photodiode, its reflectance maximum is nearly insensitive [16,37].

The study on the responsivity has focused on the range visible and infrared placing different kinds of filters on Si photodetectors. A narrowing in the responsivity spectrum of the Si photodetector was observed from the green to infrared range. Moreover, a decrease in the responsivity spectrum of the PS detectors sensitive to the color was displayed, which was compared to the responsivity spectrum of the Si photodetector without filters. The difference between both results was approximately a one magnitude order [2,37]. On the other hand, MF and BRF are used to modify the responsivity of a Si photodetector in the orange range, where the photodetector’s responsivity with a filter was stronger. It reduced the undesired effects in the responsivity spectrum [16,43].

Furthermore, it has been reported that below 500 nm, no photocurrent is detected due to the low responsivity of a silicon photodetector. Its modulation using UV filters cannot be achieved because the absorption on PS is dominant from the blue to UV range [37]. Thus, it is not possible to modulate the responsivity of broadband photodetectors in that part of the electromagnetic spectrum. To address this limitation, we employed dry oxidation to decrease the absorption losses in the UV-A range. The transmission improvement in the porous Si-SiO_2_ UV filters allowed us to modulate the responsivity of a commercial broadband photodetector.

## 4. Conclusions

In this work, porous Si-SiO_2_ UV filters were manufactured based on Si and SiO_2_. We fabricated both symmetric and antisymmetric filters in the blue region, where the antisymmetric structures gave us better results. This result was because we tried to reduce the number of layers to obtain porous silicon MFs as close as possible to the short wavelength region. Furthermore, we noticed during the dry oxidation that the number of layers had an enormous influence on the growth of SiO_2_ into the porous silicon structure. This is because large structures can be transformed entirely in porous SiO_2_. Besides, the presence of a higher quantity of silicon would cause absorption losses in the porous silicon structure; therefore, it could not be possible to obtain porous SiO_2_ MF in the UV range.

The filters were placed on quartz substrates to obtain their transmission and reflection spectra. First, we observed the onset of a localized mode in the blue region in unoxidized MFs, and second, we observed an optical shift of the localized mode location towards small wavelengths (UV region). The shift was due to the oxidation process. The ultimate consequence of the oxidation procedure was the phase change of the PS skeleton, which was converted into SiO_2_. Consequently, the refractive index decreased and the extinction values also decreased, although more drastically. The refractive index decrease is the origin of the localized mode location shift, whereas the extinction value decrease is related to a severe light absorption decrement, which means less optical losses.

The filters on quartz substrates were placed on a commercial photodetector. The light coming from a Xenon lamp was filtered out by the filters and changed the reflection at the top photodetector surface, achieving modulation of the photodetector responsivity. As a result, the spectral responsivity obtained matched the UV filter transmission spectrum. The filters’ spectral responses were modified by applying different oxidation flows in the oxidation process. When a high flow was used, the transmission in the localized mode wavelength decayed. We have shown that the photodetector became more selective in the UV range using porous UV Si-SiO_2_ filters, where an average responsivity peak arose due to the localized mode of the microcavity. The position and amplitude of the localized mode microcavity can be manipulated and tuned in the UV range, by applying different oxygen flows during dry oxidation. In this work, we demonstrated that the responsivity of a commercial photodetector could be modulated using porous Si-SiO_2_ UV filters. Moreover, the responsivity peak may be fully tunable, depending on the filter design from UV-A to near-infrared. In the future, the modulated responsivity could be enhanced using Rugate filters, and these filters could be implemented or integrated into Si-based photodetectors, thereby achieving better sensitivity to UV light. Rugate filters could exhibit a unique maximum peak in all the detection ranges of the UV photodetector. It has been reported that Rugate filters have a narrow PBG and the sidelobes on each side of these filters are smaller than other kinds of filters [29].

Other possible applications of these MFs are to embed quantum dots and liquid crystals inside porous Si-SiO_2_ UV filters to match their emission spectra with the microcavity localized mode. In this way, we would enhance and modulate the photoluminescence in the UV range by experimentally tuning the localized mode of the microcavity. Porous Si-SiO_2_ UV filters may be used as antireflection coatings [9,44] to enhance the efficiency and photocurrent in solar cells. This is because the localized mode of the microcavity shows a maximum amplitude of 70% in the UV range, whereas, in the VIS range, more than 80% of the light is transmitted. Decreasing reflection and increasing transmission in the UV filters could be raised by the efficiency of solar cells in the high-energy range. It could be achieved if a UV filter attains high transmission close to 100%.

One significant contribution of this work is that our microcavities are based on silicon solely. This contribution is important because up to now, silicon photodiodes and solar cells can only work in the UV region. However, silicon photodetectors have a broad response, and our UV microcavities can make them more selective in that range. In the future, the possibility of integrating these UV filters along with porous silicon photodetectors may be a reality, as well as UV light-emitting devices based only on silicon and silicon dioxide technology. Moreover, as shown above, similar approaches to modulate the responsivity of a photodetector have been proposed. Nevertheless, to the best of our knowledge, this is the first time that it has been done within the UV range.

What is the advantage of using porous SiO_2_ compared to other materials with similar optical properties? Some authors have reported that several fluorides with bandgap energies around 10 eV are suitable for filtering applications in the UV wavelength range, even below 200 nm. An example is a mixture of lanthanum fluoride (LaF_3_) and magnesium fluoride (MgF_2_), which have been used as high reflectance coatings. They showed strong absorption in the VIS and UV range, as well as a maximum reflectance higher than 90% at 180 nm, while their transmittance was lower than 2%. Their refractive indexes were found in the range from 1.41 to 1.80. High reflectance coatings have been produced by ion-assisted deposition, ion-beam sputtering, and electron-beam evaporation. However, several difficulties exist in these materials due to optical losses, mechanical stress of the thin films, small refractive index difference between both materials, and the optical inhomogeneity of LaF_3_ films [45,46].

On the other hand, many oxides are an essential class of coating materials, which react at high temperatures. HfO_2_ (hafnium oxide) and ZrO_2_ (zirconium oxide) are examples of transparent oxides, where HfO_2_ films are produced by electron-beam evaporation, reactive sputtering, and ion-assisted deposition. ZrO_2_ films are prepared by electron-beam evaporation, which showed strong inhomogeneity of the refractive index. HfO_2_ and ZrO_2_ are common materials used to manufacture UV filters due to their transparency, low-absorption, and high refractive index in that optical range. A long pass edge filter composed of 91 layers of HfO_2_/SiO_2_ deposited on a fused silica substrate by ion-assisted deposition (IAD) has been reported. The total thickness of the coating was around 3 µm [47,48,49]. The 50% transmission cut-on edge of this particular filter was at 256 nm, and the transmission in the passband average above 90% was from 260 nm to 1200 nm. Moisture stable HfO_2_ films and stacks containing HfO_2_ and SiO_2_ can be deposited at lower substrate temperatures (100 °C to 130 °C) using an argon oxygen gas mixture IAD process. Most thin-films deposited without ion-assistance are porous and sensitive to moisture going in and out of the voids, thereby causing an apparent shift in the refractive index depending on the relative humidity [50]. HfO_2_ films obtained by ion-assisted electron-beam evaporation showed low-absorption, with a refractive index of 2.19. In non-ion-assisted, the refractive index was 2.06, and the absorption coefficient was 0.006 and 0.003 for ion-assisted and non-ion-assisted [51]. ZrO_2_ films have been deposited by magnetron sputtering at different argon partial pressure values, where the films of ZrO_2_ showed a maximum transmission of 80% from 300 to 800 nm. This material has a high refractive index and low absorption above 240 nm to the IR range (below 8 mm) [52]. Oxide films are also widely used in multilayers systems, such as cold light mirrors, heat-reflecting filters, color separators, narrow band interference filters, and laser coatings. ZrO_2_ and HfO_2_ have disadvantages; for instance, they form uneven surfaces during evaporation, which often cause inhomogeneous thickness distributions and inferior thickness reproducibility. It has been investigated that no pure oxides exist with refractive indices of about 2.2. Available mixtures of silver (Ag)–silicon dioxide (SiO_2_) can be found, which have been used as thin-film bandpass filters for the UV range obtained by radiofrequency sputtering. In this filter, the suppression of undesired visible and infrared parts of the spectrum was achieved. Some structures applicable for bandpass filtering in the UV have high transmission in the passband but a limited range of out-band blocking. An additional blocking component such as monolithic deposited silver has been used to remove unwanted out-of-band radiation. However, this also reduces the overall transmission through the filter. Some pairs of Ag/SiO_2_ layers and a layer of silica were added to serve as an antireflection coating, where the band-pass filter was designed with the maximum transmission in the UV-A range and with a resonance at 320 nm. The use of transparent metals can ensure a transmission decrease of several orders of magnitude in the visible and infrared wavelength range, at the same time, fully preserving transparency in the ultraviolet spectrum [53].

Solar-blind deep-UV band-pass filters based on a mixture of aluminum (Al) and SiO_2_ have been developed. The filters showed a 27% transmission peak at 290 nm, a band-pass from 250 to 350 nm, and a rejection ratio to visible light of 20 dB, where the peak of transmission could be tuned by adjusting the metal nano-grind dimensions [54]. An alternative approach to solar-blind UV detection is to integrate Si-based photodetectors with solar-blind UV-pass filters to reject visible and IR light in the solar spectrum. Moreover, the designed and fabricated filters are fully applicable for the enhancement of UV silicon detectors since one of the problems with the use of silicon photodetectors is the avoidance of the visible and infrared components of radiation. Under standard conditions, it often exceeds the UV components by three to four orders of magnitude, thereby completely masking the useful signal. A straightforward way to avoid the problem is to use bandpass filters for the UV strong rejection of unwanted visible and infrared radiation in the range of the detector sensitivity. However, a disadvantage of Ag/SiO_2_ and Al/SiO_2_ structures is that they present reduced transmission (less than 50%) in the UV range due to the absorption of the Ag and Al.

Moreover, dielectric interference filters have also been demonstrated for solar-blind UV applications. However, this type of filter requires a thick stack of multiple dielectric materials layers that must be deposited with precise thickness and uniformity control, with high quality over a very long deposition time. This inevitably increases production costs and reduces the uniform area. The filters also need accurate control of pressure and temperature.

In our work, dielectric interference filters in the UV were achieved by electrochemical etching, followed by two-stage dry oxidation. Our method was easy, cheap, and fast to fabricate compared to the methods mentioned before. The thickness of each layer that made up the UV dielectric microcavity was precise and uniform, and the fabrication time of these dielectric microcavities was shorter than other methods. Besides, physical vapor deposition infrastructure is expensive, and ion-assisted deposition is a costly and slow technique. Additionally, dielectric oxide films do not show sufficient thermal stability because the structure of the oxide films is easily converted from amorphous to polycrystalline and it reacts with the Si substrate.

To conclude, the porous Si-SiO_2_ UV filters (MF2, MF3, and MF4) can be applied as UV hot mirrors or UV bandpass filters since they display more than 70% of the transmitted light in the UV-VIS range. Meanwhile, the MF5 filter can be used as a UV blocking filter since it cuts off more UV light than other filters, and it shows a maximum transmission amplitude of 80% in the VIS-IR.

## Figures and Tables

**Table 1:** Theoretical values of porosity before and after dry oxidation, thickness, Si fraction, and oxide fraction of five microcavities.

Filter	Porosity (%)	$f_{Si}\;(\%)$	$f_{ox}\;(\%)$	$p_{ox}\;(\%)$	Average Thickness (nm)
MF1	36	64	---------	---------	$d_{H}=35$
	57	43			$d_{L}=68.7$
MF2	---------	0.23	98.51	1.27	$d_{H}=\;$46.5
		0.25	78.86	20.89	$d_{L}=78.0$
MF3	---------	0.78	97.88	1.34	$d_{H}=\;$47.9
		0.15	79.02	20.83	$d_{L}=78$.8
MF4	---------	1.42	97.14	1.44	$d_{H}=\;$47.7
		0.22	78.91	20.87	$d_{L}=78.8$
MF5	---------	2.92	95.41	1.67	$d_{H}=\;$47.5
		0.01	79.23	20.76	$d_{H}=78$.1

## Supplementary Materials

The following are available online at https://www.mdpi.com/2079-4991/10/2/222/s1.
