# Supplementary material for: Porous Si-SiO2 UV Microcavities to Modulate the Responsivity of a Broadband Photodetector
Source: Nanomaterials (Basel). 2020 Jan 28;10(2):222. doi: 10.3390/nano10020222 (PMC7075018; doi:10.3390/nano10020222)
Supplement: Supplementary file 1 [file nanomaterials-10-00222-s001.pdf]

*nanomaterials*

MDPI

*Article*

# Porous Si-SiO2 UV Microcavities to Modulate the Responsivity of a Broadband Photodetector

**María R. Jimenéz-Vivanco [1], Godofredo García [1], Jesús Carrillo [1], Francisco Morales [2], Antonio Coyopol [1], Miguel Gracia [3], Rafael Doti [4], Jocelyn Faubert [4] and J. Eduardo Lugo [4,*]**

[1] Centro de Investigación en Dispositivos Semiconductores, ICUAP, BUAP, Ciudad Universitaria, Puebla Pue. C.P. 72570, Mexico; jimenezvmr10@gmail.com (M.R.J.-V.); godgarcia@yahoo.com (G.G.); jecarril@siu.buap.mx (J.C.); acoyopol@gmail.com (A.C.)

[2] Centro de Investigaciones en óptica, Ed. H-1307, Col. Lomas del campestre, León Gto. C.P. 37150, Mexico; fcomm9@gmail.com

[3] IFUAP, Benemérita Universidad Autónoma de Puebla, Ed. IF2, Col. San Manuel, Puebla C.P. 72570, Mexico; gracia@ifuap.buap.mx

[4] Faubert Lab, École d'optométrie, Université de Montréal, Montréal, QC H3T 1J4, Canada; rafael.doti@gmail.com (R.D.); faubert.umontreal@gmail.com (J.F.)

* Correspondence: eduardo.lugo@gmail.com; Tel.: +1-514-343-6111 (ext. 1685)

## Theory/ calculation

The transfer matrix was used to calculate the theoretical reflection, transmission, and absorption spectra of PS and porous Si-$SiO_2$ microcavities filters. The transfer matrix is very well-known [1]; it can be resolved for N numbers of layers, and it can be written as

$$\begin{pmatrix} M_{11} & M_{12} \\ M_{21} & M_{22} \end{pmatrix} = D_0^{-1}\left[\prod_{l=1}^{N} D_l P_l D_l^{-1}\right] D_s \qquad (1)$$

Where $M_{11}$, $M_{12}$, $M_{21}$, $M_{22}$ are matrix elements; they are fundamental to describe how electromagnetic radiation propagates through a periodic medium; $D_l$ is the dynamical matrix of PS, $D_0$ is the dynamical matrix of air and $D_s$ is the dynamical matrix of the substrate and $P_l$ is the propagation matrix. In these simulations, complex dielectric constants (refractive index and extinction coefficients) of PS were taken into account.

The effective medium approximation Maxwell-Garnett was applied to calculate the effective dielectric constant (refractive index and extinction coefficient) of PS layers. From Maxwell-Garnett, knowing the volume fraction of Si and void, the effective dielectric constant of PS layers was deduced. The equation for two mediums is expressed as follows:

$$\frac{\varepsilon_{PS}-\varepsilon_{Si}}{\varepsilon_{PS}+\varepsilon_{Si}} = P\frac{\varepsilon_{air}-\varepsilon_{Si}}{\varepsilon_{air}+\varepsilon_{Si}} \qquad (2)$$

Where $P$ (porosity) is the air fraction of the non-oxidized PS layers, $\varepsilon_{air}$ is the dielectric constant of the air; $\varepsilon_{Si}$ is the dielectric constant of Si, and $\varepsilon_{PS}$ represents the effective dielectric constant of PS.

The effective dielectric constant of porous Si-$SiO_2$ layers was obtained using a model proposed by J. E. Lugo. The three components model (Si, $SiO_2$, and air) takes into account the presence of $SiO_2$ and its network expansion that occurs within the PS structure, due to the increase of $SiO_2$ in the porous matrix of SP [2].

The refractive index for the three components can be obtained using the following equation:

$$\frac{\varepsilon_{PS}-\varepsilon_{Si}}{\varepsilon_{PS}+\varepsilon_{Si}} = P^*\frac{\varepsilon_1^*-\varepsilon_3^*}{\varepsilon_1^*+\varepsilon_3^*} \qquad (3)$$

Where $\varepsilon_1^*$ and $\varepsilon_3^*$ contain the dielectric constant values of silicon dioxide ($SiO_2$) and air, $P^* = \frac{P_{ox}}{\beta}$ and $P_{ox}$ is the porosity after the dry oxidation.

The parameter $\beta$ is represented by:

$$\beta = \left[\frac{1-0.55x}{1+0.45x}\right]^2, \qquad (4)$$

where $x$ is a dimensionless oxidation parameter.

The constants 0.55 and 0.45 in equation (4) are related to oxide growth in Si. Therefore, an oxide layer grows 55% above the Si wafer surface and 45 % below the original surface.

The porosity for a system of three components can be calculated as

$$P_{ox} = P[1 - 0.55x]^2 \quad (5)$$

Hence the Si is consumed as the oxide grows, and a volume expansion occurs during oxidation [3]. We can obtain the oxide fraction ($f_{ox}$) after dry oxidation as

$$f_{ox} = P[2x - 0.1x^2] \quad (6)$$

Therefore, the Si fraction $f_{Si}$ can be calculated using the following equation:

$$f_{Si} = 1 - P_{ox} - f_{ox} \quad (7)$$

These models consider a homogeneous medium with an effective complex dielectric function.

We used the dispersion relation to obtain the theoretical photonic band structure of the PS microcavities filters; it has been linked to the matrix elements, and it can be written as

$$K(\beta, \omega) = \frac{1}{d}\cos^{-1}\left[\frac{1}{2}(M_{11} + M_{22})\right] \quad (8)$$

In this equation, $K$ is the Bloch wave number, $\beta$ is the off-axis wave vector component, $d$ is the spatial period that equals the addition of individual thicknesses, and $\omega$ is the frequency. The formation of forbidden bands where the propagation of photons is not allowed is produced by the periodic variation of PS dielectric constants. The most common example of the photonic bandgap structures is BRFs. A microcavity has one or more photonic states where the photons can be localized in the defect region at some frequency and energy. Photonic modes are classified as defects or localized modes and extended modes [4]. We have taken the absolute value of the refractive index and its wavelength average within the whole experimental spectral range for PBG structure calculations; it was necessary because this theory does not apply to materials that present wavelength dispersion.

To find the theoretical frequency of a localized mode inside the bandgap, we use the method of the optical transfer matrix (OTM) [4]. It is closely related to the transference matrix method, and it directly allows the description of the connection between adjacent regions by continuity conditions at the endpoints. It can be calculated at separate regions, so the optical properties of BRFs with N periods and a defect are determined by considering a superposition of a right-traveling plane wave and a left-traveling plane wave.

Thus, the function $\gamma_N = \frac{M_{11}-M_{21}}{M_{22}-M_{12}}$ relates to the matrix elements. It has a relationship to the function $\kappa(\omega)$, which depends on the frequency; and it can be written as:

$$\kappa(\omega) = k_d \mathrm{Im}\left(\frac{1+\gamma_N}{1-\gamma_N}\right), \quad (9)$$

where $k_d$ is the z component of the wavevector for the first layer (defect layer), where the z-axe is taken along the normal direction to the layers (Figure 1). We can obtain the variation of the localized mode frequencies for an antisymmetric or symmetric state. In the antisymmetric case these frequencies can be expressed as:

$$h(\omega) = -k_d \cot(k_d L) \text{ for antisymmetric states} \quad (10)$$

Here $L$ is the half-length of the defect layer, and $k_d$ is the z component of the wavevector as it was mentioned before. We consider only the antisymmetric case because our microcavity design is antisymmetric and the condition where the functions $\kappa(\omega)$ and $h(\omega)$ intersect gives the localized mode frequency. For the localized mode calculation, we used the wavelength average of the complex refractive index absolute value.

We used the Beer-Lambert law to get the MCs experimental absorbance spectrum on quartz substrates; this equation can be expressed as

$$A = 2 - \log_{10} 1/T \quad (11)$$

Where A is the absorbance, and $T$ represents the transmission. This equation is related to the light intensity that passes through the microcavity[5], where part of the light is either absorbed or transmitted.

1. P., Y. *Optical Waves in Layered Media*; John Wiley & Sons, Inc.: New York, USA, 2005.
2. Lugo, J.; Lopez, H.; Chan, S.; Fauchet, P. Porous silicon multilayer structures: A photonic band gap analysis. *Journal of applied physics* **2002**, *91*, 4966-4972, doi:10.1063/1.1461898.
3. Sze, S.M. *Semiconductors Devices Physics and Tecnology*, 2nd ed.; John Wiley & Sons, Inc.: New York, 2002.
4. Van Groesen, E.; Sopaheluwakan, A.; Andonowati. Direct characterization of states and modes in defect grating structures. *Journal of nonlinear optical physics & materials* **2004**, *13*, 155-173, doi:10.1142/S0218863504001839.
5. Dandin, M.; Abshire, P.; Smela, E. Optical filtering technologies for integrated fluorescence sensors. *Lab on a Chip* **2007**, *7*, 955-977, doi:10.1039/B704008C.
